# Full-dimensional theoretical description of vibrationally resolved valence-shell photoionization of H_2_O

**DOI:** 10.1063/1.5106431

**Published:** 2019-09-11

**Authors:** Selma Engin, Jesús González-Vázquez, Gianluigi Grimaldi Maliyar, Aleksandar R. Milosavljević, Taishi Ono, Saikat Nandi, Denys Iablonskyi, Kuno Kooser, John D. Bozek, Piero Decleva, Edwin Kukk, Kiyoshi Ueda, Fernando Martín

**Affiliations:** 1 Departamento de Química, Módulo 13, Universidad Autónoma de Madrid, 28049 Madrid, Spain; 2 Institute of Multidisciplinary Research for Advanced Materials, Tohoku University, Sendai 980-8577, Japan; 3 Synchrotron SOLEIL, L'Orme des Merisiers, Saint-Aubin, BP 48, 91192 Gif-sur-Yvette Cedex, France; 4 Department of Physics and Astronomy, University of Turku, 20014 Turku, Finland; 5 Institute of Physics, University of Tartu, EST-50411 Tartu, Estonia; 6 Dipartimento di Scienze Chimiche e Farmaceutiche, Universitá di Trieste and IOM-CNR, 34127 Trieste, Italy; 7 Instituto Madrileño de Estudios Avanzados en Nanociencia (IMDEA-Nanociencia), Cantoblanco, 28049 Madrid, Spain; 8 Condensed Matter Physics Center (IFIMAC), Universidad Autónoma de Madrid, 28049 Madrid, Spain

## Abstract

We have performed a full-dimensional theoretical study of vibrationally resolved photoelectron emission from the valence shell of the water molecule by using an extension of the static-exchange density functional theory that accounts for ionization as well as for vibrational motion in the symmetric stretching, antisymmetric stretching, and bending modes. At variance with previous studies performed in centrosymmetric molecules, where vibrationally resolved spectra are mostly dominated by the symmetric stretching mode, in the present case, all three modes contribute to the calculated spectra, including intermode couplings. We have found that diffraction of the ejected electron by the various atomic centers is barely visible in the ratios between vibrationally resolved photoelectron spectra corresponding to different vibrational states of the remaining H_2_O^+^ cation (the so-called *v*-ratios), in contrast to the prominent oscillations observed in K-shell ionization of centrosymmetric molecules, including those that only contain hydrogen atoms around the central atoms, e.g., CH_4_. To validate the conclusions of our work, we have carried out synchrotron radiation experiments at the SOLEIL synchrotron and determined photoelectron spectra and *v*-ratios for H_2_O in a wide range of photon energies, from threshold up to 150 eV. The agreement with the theoretical predictions is good.

## I. INTRODUCTION

Third- and fourth-generation synchrotron radiation (SR) in combination with high-resolution detection of photoelectron energies allows one to vibrationally resolve photoelectron spectra of diatomic and small polyatomic molecules.^1–20^ The analysis of the measured ratios between photoelectron spectra associated with different vibrational states of the remaining molecular cation (for short, *v*-ratios) as a function of photon energy usually reveals structures that are difficult to identify in the corresponding cross sections, which decay exponentially with photon energy. In particular, shape and Feshbach resonances lying up to several tens of electron volts above the ionization threshold appear in the *v*-ratios as very pronounced peaks that allow for an easy identification.^8,17,19–22^ Also, interferences due to multicenter photoelectron emission^13,14^ (analogous to Young's multislit interferences) or to diffraction of the (initially but not necessarily localized) photoelectron by the surrounding molecular environment^13,15–18^ lead to pronounced oscillations whose amplitude and frequency thus contain structural information on the molecule.

In the case of diatomic molecules,^13,19,23,24^ the analysis of the measured *v*-ratios is relatively simple, since only one vibrational degree of freedom is available. In K-shell photoionization of small symmetric polyatomic molecules from their central atom, such as CH_4_, CF_4_, and BF_3_, the situation is similar since all chemical bonds are equally affected by the loss of the electron initially localized in the central electron and the molecular symmetry is barely affected. Consequently, the symmetric stretching mode entirely dominates the vibrational progressions observed in the photoelectron spectra.^14–18,20^ This is also the case of inner-valence photoionization of CH_4._^25^ Thus, theoretical modeling of the vibrationally resolved photoelectron spectra is relatively simple, since dipole transition matrix elements only need to be evaluated along a single nuclear degree of freedom. This simplicity is crucial to extract structural information, such as bond distances, directly from the frequency of the observed oscillations.^13,26^ It also explains why *v*-ratios resulting from H_2_ photoionization have recently permitted to reconstruct the subfemtosecond time evolution of the electron wave packet that arises from H_2_ autoionization, thus forgoing ultrashort light pulses.^19^

Early K-shell photoionization experiments performed in the less symmetric water molecule indicate that the vibrational progression observed in the photoelectron spectra is mainly due to the bending mode^27^ so that a one-dimensional theoretical treatment of molecular vibrations is still appropriate. However, in valence shell photoionization of this and less symmetric molecules, where the electron is ejected from delocalized molecular orbitals, one can expect substantial changes in the chemical bonds and, therefore, in the molecular symmetry, as such changes will not be symmetrically distributed in space. One can thus expect that vibrational progressions associated with different normal modes will show up in the photoelectron spectrum. Correspondingly, theoretical modeling will be much more cumbersome, not only because dipole transition elements must be evaluated in a multidimensional grid of molecular geometries, which would increase exponentially the computational cost, but also because the different normal modes can combine and couple to each other, thus leading to a very complex vibrational structure. In this scenario, extraction of structural information from the measured *v*-ratios is not easy and, in fact, to our knowledge, it has not been achieved to date.

Therefore, in order to make progress in the interpretation of valence-shell vibrationally resolved photoionization spectra of such molecules in the wide range of photon energies that are covered in a typical experiment, one has to improve the existing theoretical methods to account for the full dimensionality of the problem. In this work, we have extended the static-exchange density functional theory (DFT) method, successfully used in the past to describe (i) vibrationally unresolved photoionization spectra within the fixed nuclei approximation and (ii) K-shell vibrationally resolved photoionization spectra of diatomic and central symmetric molecules by just including the symmetric stretching mode, to account for the full dimensionality of the problem. The method is used here to obtain valence-shell vibrationally resolved photoelectron spectra of the water molecule. Static-exchange DFT has been shown to be very reliable to describe features in the ionization continuum due to a single active electron (see, e.g., Ref. 28 and references therein), in particular, shape resonances, interference, and diffraction effects. To check the validity of the method, we have measured the corresponding *v*-ratios at the Pleiades line of the SOLEIL synchrotron. These measurements extend earlier high-resolution threshold photoelectron spectroscopy studies of H_2_O (see Ref. 29 and references therein) with which a direct comparison with the present theoretical calculations is difficult. The good agreement between theory and the present experiment validates the theoretical treatment and shows that the bending and the two stretching modes (symmetric and antisymmetric) play a crucial role in the measured spectra. These spectra also reflect the couplings between some of these modes.

This paper is organized as follows: in Secs. II and III, we describe the theoretical and experimental methods, respectively. In Sec. IV, we present the main results of the present work, in particular, the calculated and measured *v*-ratios, by putting emphasis on the new physics that arises by considering all vibrational degrees of freedom and on the complexity that this introduces in identifying clear diffraction patterns for structural determination. In Sec. V, we summarize the main conclusions of our work and suggest further research directions in H_2_O and other molecules.

## II. THEORETICAL METHODS

### A. Cross sections

We have evaluated vibrationally resolved photoionization cross sections $\sigma_{\alpha }\left(v\prime ,\omega \right)$ to first order of perturbation theory,

$$\sigma_{\alpha }\left(v\prime ,\omega \right)=\frac{4\pi^{2}\omega }{3\hbar c}a_{0}^{2}\sum_{\eta }\sum_{l_{\eta }}{\left|T_{\alpha_{\eta }l_{\eta }v^{\prime }}\left(\varepsilon \right)\right|}^{2}.$$ (1)

In this equation, $T_{\alpha_{\eta }l_{\eta }v\prime }$ is the ionization amplitude associated with leaving the residual cation in the vibrational state $v\prime =(v_{\mathrm{ss}}^{\prime },v_{b}^{\prime },v_{\mathrm{as}}^{\prime })$, where $v_{\mathrm{ss}}^{\prime },\;v_{b}^{\prime }$, and $v_{\mathrm{as}}^{\prime }$ are the symmetric stretching, bending, and antisymmetric stretching vibrational quantum numbers, respectively, *ω* is the photon energy, *α* denotes the electronic state of the residual ion, *η* is the symmetry of the final electronic state, *ε* is the energy of the photoelectron, and *l_η_* is its angular momentum. Within the Born-Oppenheimer (BO) and dipole approximations, the ionization amplitude is written as

$$T_{\alpha_{\eta }l_{\eta }v\prime }\left(\varepsilon \right)=\int \chi_{M,v_{0}}^{*}\left(Q\right)\mu_{\alpha_{\eta }l_{\eta }}\left(\varepsilon ,Q\right)\chi_{M_{\alpha }^{+},v\prime }\left(Q\right)dQ,$$ (2)

where $\mu_{\alpha_{\eta }l_{\eta }}$ is the dipole-transition matrix element between the initial electronic state, *ψ*_0_, and the final electronic continuum state, $\psi_{\alpha_{\eta }l_{\eta }}$, of H_2_O,

$$\mu_{\alpha_{\eta }l_{\eta }}\left(\varepsilon ,Q\right)=\int \psi_{0}(q)[\epsilon_{p}\cdot q]\psi_{\alpha_{\eta }l_{\eta }}(q)dq,$$ (3)

where $\chi_{M,v_{0}}$ is the initial vibrational state and $\chi_{M_{\alpha }^{+},v\prime }$ is the final vibrational state. In these equations, **q** and **Q** represent the sets of electronic and vibrational coordinates, respectively.

### B. Electronic structure calculations

We have evaluated the electronic continuum state $\psi_{\alpha_{\eta }l_{\eta }}$ by using the static-exchange density functional theory (DFT).^30,31^ This method has been shown to provide accurate photoionization cross sections for a large variety of molecules within the fixed nuclei approximation (see, e.g., Refs. 32–34) and by including the vibrational degree of freedom in diatomic molecules^13,23,24,26^ and the dominant stretching one in small polyatomic molecules.^14–18,20^ In this work, we have extended this method to include all vibrational degrees of freedom of H_2_O. More specifically, we have evaluated the dipole-transition matrix element $\mu_{\alpha_{\eta }l_{\eta }}$ in a three-dimensional grid for the symmetric stretching, antisymmetric stretching, and bending vibrational modes. This grid accounts for 1053 different geometries. For each geometry, we have adopted the following procedure. The initial Kohn-Sham orbitals have been obtained from a standard linear combination of atomic orbitals (LCAO)-DFT calculation by using the Amsterdam Density Functional (ADF) code^35^ with a Vosko-Wilk-Nusair (VWN) functional for exchange and correlation and a double zeta-polarization (DZP) basis set. We have then defined a single-electron Kohn-Sham Hamiltonian ${\hat{H}}_{\mathrm{KS}}$ by using the so-called transition state (TS) DFT density relative to the ionized orbital, i.e., decreasing its occupation by half an electron, as originally proposed by Slater. It has been shown that VWN in conjunction with TS gives a good alternative choice for computing photoionization cross sections.^36^ The corresponding Schrödinger equation has been solved by expanding the wave function in a basis of symmetry adapted products of B-spline functions and real spherical harmonics. Two different kinds of expansions are considered: (i) a one-center expansion (OCE) located at the oxygen nucleus with B-splines defined in a large spherical box of 30 a.u., which allows us to properly represent the long range oscillatory behavior of the continuum wave function, and (ii) two off-center expansions, namely, at the two hydrogen nuclei, with B-splines defined in small spherical boxes of 0.8 a.u., which is necessary to accurately describe the cusps of bound state wave functions and to accelerate convergence with angular momentum. The chosen radii lie within the usual range of values used in most previous applications of the static-exchange DFT method (see Refs. 28 and 33, and references therein), and the parameters chosen ensure effective convergence of the reported cross sections. Bound states are evaluated by diagonalizing ${\hat{H}}_{\mathrm{KS}}$ and continuum states by block inverse iteration^37,38^ for each given photoelectron energy. The latter procedure ensures that the resulting continuum wave functions satisfy the proper scattering boundary conditions. Electronic dipole matrix elements were computed for 201 photoelectron energies, in a basis of 303 B-splines, and symmetry adapted spherical harmonics up to *l *=* *12.

Three-dimensional potential energy surfaces for the neutral molecule and the cation were obtained by performing CAS(10,7) and CAS(9,7) calculations, respectively. In the case of the cation, a state average of 3 states was considered, SA3-CAS(9,7). These calculations were performed with the MOLPRO code,^39^ in which all configurations (subject to spin restrictions) considering all electrons in the 1s, 2s, and 2p orbitals of the O atom and the 1s orbital of the two H atoms were included. These orbitals were represented by a standard cc-pvtz (polarized valence triple zeta) basis^40^ of localized Gaussian functions. Table I shows the calculated OH distance and HOH angle for the equilibrium geometries of the ground state of the neutral molecule and the ^2^B_1_, ^2^A_1_, and ^2^B_2_ states of the H_2_O^+^ cation. For comparison, the results of more elaborate CASPT2 calculations we have performed with the same basis and other accurate theoretical and experimental results reported previously are given. As can be seen, the general agreement is reasonably good.

**TABLE I.** Bond distance (*d*_OH_) and angle (∠HOH) for the equilibrium geometries of the ground state of the neutral molecule and the ^2^B_1_, ^2^A_1_ and ^2^B_2_ states of the H_2_O^+^ cation. FPD: theoretical results from Ref. 41 using the Feller-Peterson-Dixon approach. Experimental values taken from Refs. 42–45. For simplicity, ranges of these values provided by the latter references are given.

		CASSCF	CASPT2	FPD^41^	Exp.^42–45^
H_2_O(^1^A_1_)	*d*_OH_ (Å)	0.9637	0.9578	0.9557	0.95799
	∠HOH (deg)	102.61	103.88	104.51	104.40
H_2_O^+^(^2^B_1_)	*d*_OH_ (Å)	1.005	1.0002	0.9985	0.999–1.03
	∠HOH (deg)	107.70	109.04	109.47	109.3–110.5
H_2_O^+^(^2^A_1_)	*d*_OH_ (Å)	0.9871	0.9876	0.9875	…
	∠HOH (deg)	176.72	176.46	180.0	…
H_2_O^+^(^2^B_2_)	*d*_OH_ (Å)	1.14121	1.1240	1.1256	…
	$\hat{\text{HOH}}\;(\deg)$	54.56	57.13	57.61	…

It is important to emphasize that the evaluation of vibrationally resolved photoionization cross sections requires the computation of PESs for both the neutral molecule and the cation over a thousand of different geometries. In addition, one is forced to calculate the true molecular continuum wave functions for all these geometries, since methods based on plane wave or Coulomb wave descriptions of the ionized electron^46^ would miss shape resonances and diffraction effects as those sought for in the present work. Thus, using a higher level of theory for the description of the electronic structure of the neutral molecule and the cation would make calculations extremely expensive.

### C. Vibrational wave functions

We have solved the three-dimensional vibrational Schrödinger equation for the ground electronic state of the neutral molecule and the cation by expanding the vibrational wave function in a basis of products of one-dimensional wave functions, $\left\{\phi_{i}^{s}(Q_{s})\phi_{j}^{b}(Q_{b})\phi_{k}^{a}(Q_{a})\right\}$, as follows:

$$\chi_{\alpha ,v\prime }\left(Q\right)=\sum_{i}^{N_{s}}\sum_{j}^{N_{b}}\sum_{k}^{N_{a}}c_{\mathrm{ijk}}^{\alpha ,v^{\prime }}\phi_{i}^{s}(Q_{s})\phi_{j}^{b}(Q_{b})\phi_{k}^{a}(Q_{a}),$$ (4)

where $\phi_{i}^{s}(Q_{s}),\;\phi_{j}^{b}(Q_{b})$, and $\phi_{k}^{a}(Q_{a})$ are the one-dimensional ground-state harmonic-oscillator wave functions for the symmetric stretching, bending, and antisymmetric stretching modes and *Q_s_*, *Q_b_*, and *Q_a_* are the corresponding normal mode coordinates evaluated in cartesian coordinates, **Q** = *Q_s_Q_b_Q_a_*. We have checked that, for the low vibrational quantum numbers that are required for the present calculations (see below), convergence of the vibrational wave functions associated with the electronic ground state of neutral and ionized water is achieved with *N_s_* = *N_b_* = *N_a_* = 10, i.e., 1000 terms in the expansion. The one-dimensional vibrational functions have been represented in a basis of 127, 93, and 113 grid points, respectively, including the points at the minima of the corresponding PESs.

## III. EXPERIMENTAL METHODS

The experiment was performed at the PLEIADES beamline^47–49^ at the SOLEIL national synchrotron radiation (SR) facility in France. SR was generated using an Apple II-type permanent magnet HU80 (80 mm period) undulator and monochromatized by varying groove depth, varying line spacing, 2400 l/mm, plane grating monochromator. Photoelectron spectra were recorded using a 30° wide angle lens VG-Scienta R4000 electron energy analyzer, whose detection axis is perpendicular to the storage ring plane containing the propagation direction of the SR. The angle between the polarization vector of the SR and the electron detection axis was 54.7°, excluding the photon energy dependent modulation of the cross section due to the photoelectron asymmetry *β* parameter in the dipole approximation.

The H_2_O sample had a purity of 99.995%, and it was used as provided by Messer Schweiz AG. The sample vapor at room temperature was introduced in a gas cell equipped with a series of polarized electrodes, used to minimize the effect of plasma potentials caused by the ion density gradient created along the SR propagation axis. The pressure was 4 × 10^−6^ mbar inside the experimental chamber and approximately two or three orders of magnitude higher inside the gas cell in the interaction region.

The instrumental electron energy resolution is determined by the exit slit of the monochromator (photon bandwidth) and the entrance slit and the pass energy of the electron energy analyzer. Also, the translational Doppler effect broadens the measured spectra. The spectra were measured over a range of photon energies up to 150 eV. At 90 eV, the slit widths of the monochromator and the analyzer were chosen to provide the bandwidth of about 2 meV and the energy resolution of about 5 meV, respectively. The analyzer was operated at a pass energy of 20 eV.

## IV. RESULTS

**FIG. 1.**
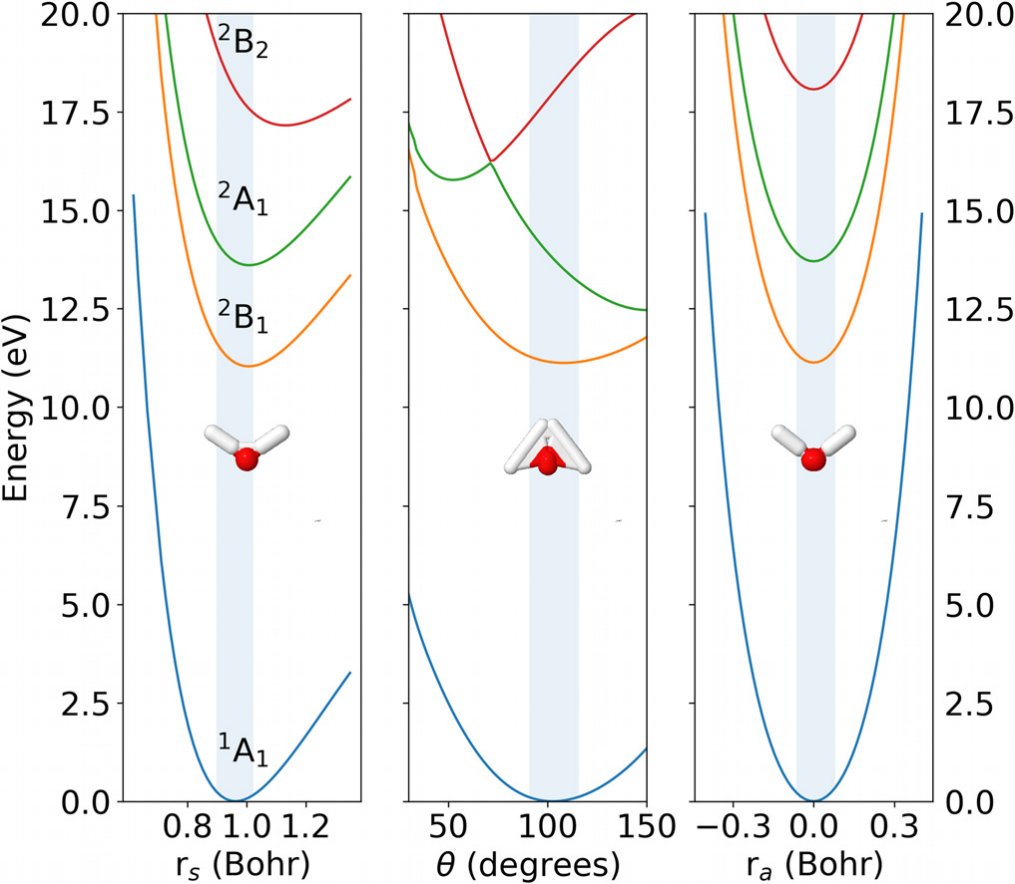
Potential energy surfaces of the ^1^A_1_ ground state of H_2_O and the ^2^B_1_, ^2^A_1_, and ^2^B_2_ states of the H_2_O^+^ cation. Only one-dimensional cuts along the symmetric stretching (left panel), bending (middle panel), and antisymmetric stretching (right panel) normal mode coordinates are shown. The energy scale is referred to the ground state energy at the equilibrium geometry. The blue shaded areas indicate the Franck-Condon region.

Figure 1 shows cuts of the potential energy surfaces of the ground state of H_2_O, ^1^A_1_, the ground state of H_2_O^+^, ^2^B_1_, and the first and the second excited states of H_2_O^+^, ^2^A_1_ and ^2^B_2_, along the symmetric stretching, bending, and antisymmetric stretching normal coordinates. As can be seen, the PESs of the H_2_O(^1^A_1_) and H_2_O^+^(^2^B_1_) states are nearly parallel along the three cuts shown in the figure so that the largest Franck-Condon (FC) overlaps between the initial (0, 0, 0) vibrational state of the neutral molecule and the final $\left(v_{\mathrm{ss}}^{\prime },v_{b}^{\prime },v_{\mathrm{as}}^{\prime }\right)$ vibrational state of the H_2_O^+^(^2^B_1_) ion must necessarily be associated with the lowest values of $v_{\mathrm{ss}}^{\prime },\;v_{b}^{\prime }$, and $v_{\mathrm{as}}^{\prime }$ (see the FC region in Fig. 1 and the FC overlaps given in Table II). Therefore, photo-ionization leading to H_2_O^+^ in its ground electronic state ^2^B_1_ can only leave the ion in bound vibrational states for all three modes. The same applies to the electronically excited H_2_O^+^(^2^A_1_) and H_2_O^+^(^2^B_2_) states for the symmetric and antisymmetric stretching modes, but not for the bending mode, as a substantial amount of H_2_O^+^ will dissociate due to the displacement of the minimum of the H_2_O^+^(^2^B_2_) state toward 180° and the presence of a conical intersection between the latter state and the H_2_O^+^(^2^A_1_) state in the FC region. Thus, for a direct comparison with the experiments presented below, we will only consider photoionization leading to H_2_O^+^ in its ground ^2^B_1_ electronic state. As this state lies very far away from the upper two states in the FC region, nonadiabatic couplings are expected to play a negligible role, thus supporting the use of the BO approximation in Eq. (2).

**TABLE II.** Energies (E in meV) and Franck-Condon (FC) factors of the vibrational states associated with the ^2^B_1_ ground electronic state of H_2_O^+^. The $v_{\mathrm{ss}}^{\prime },\;v_{b}^{\prime },\;v_{\mathrm{as}}^{\prime }$ quantum numbers refer to the dominant component in the vibrational eigenvector. Experimental energies and FC factors from Refs. 50 and 51, respectively. Reported errors in the former lie in between 0.4 and 0.5 meV.^50^

$\left(v_{\mathrm{ss}}^{\prime },v_{b}^{\prime },v_{\mathrm{as}}^{\prime }\right)$	E (meV)	E (exp.)^50^	FC factor	FC factor (exp.)^51^
(0,0,0)	0.0	0.0	0.73024	0.757 ± 0.005
(0,1,0)	179.2	174.4	0.07335	0.069 ± 0.005
(0,2,0)	353.3	344.0	0.01003	…
(1,0,0)	393.5	397.4	0.13798	0.143 ± 0.005
(0,0,1)	398.5	…	0.00000	…
(0,3,0)	522.7	506.5	0.00083	…
(1,1,0)	570.2	569.5	0.01256	0.013 ± 0.002
(0,1,1)	571.6	…	0.00000	…
(0,4,0)	689.5	…	0.00007	…
(0,2,1)	739.9	…	0.00000	…
(1,2,0)	742.3	736.0	0.00190	…
(1,0,1)	774.7	…	0.00000	…
(2,0,0)	775.9	778.6	0.01544	0.018 ± 0.002
(0,0,2)	794.9	…	0.00542	…
(0,5,0)	860.8	…	0.00001	…
(0,3,1)	903.5	…	0.00000	…
(1,3,0)	911.4	896.9	0.00018	…
(1,1,1)	945.1	…	0.00000	…
(2,1,0)	946.4	947.1	0.00102	>0.002

**FIG. 2.**
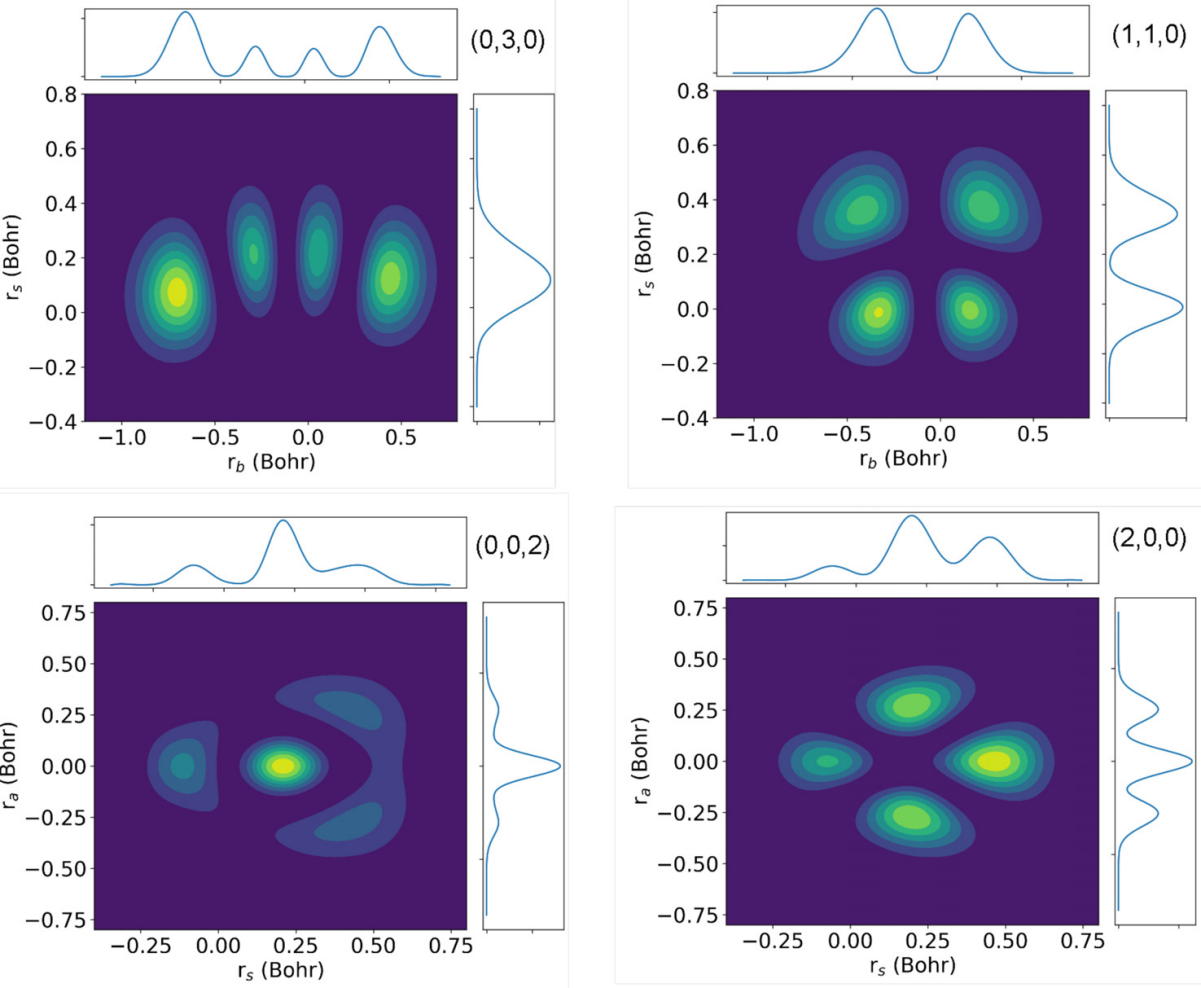
Two-dimensional density cuts of the (0, 3, 0) and (1, 1, 0) vibrational wave functions along the symmetric stretching and bending normal coordinates and of the (0, 0, 2) and (2, 0, 0) wave functions of the H_2_O^+^(^2^B_1_) state along the antisymmetric and symmetric normal coordinates. The panels also show the one-dimensional cuts that result from integrating over the corresponding coordinates.

Figure 2 shows a few vibrational wave functions associated with the H_2_O^+^(^2^B_1_) state. The wave functions for the bending and symmetric stretching modes significantly differ from those obtained from the harmonic approximation, which is not surprising in view of the important anharmonicity of the corresponding cuts in the PES shown in Fig. 1. As a consequence of this, there is not a perfect separation between the bending and symmetric stretching modes, which will thus be slightly coupled. Anharmonicities are clearly seen in the (0, 3, 0) and (1, 1, 0) wave functions when plotted along the symmetric stretching and bending normal coordinates. However, they still preserve the expected nodal structures for the corresponding harmonic modes. A paradigmatic example is the case of the quasidegenerate (0, 0, 2) and (2, 0, 0) states, whose wave functions do not exhibit the typical two-node structure for the excited mode and the no-node structure for the nonexcited modes. This is the consequence of the strong interaction between the two modes. Table II shows the vibrational energies of the final vibrational states and the FC overlaps between the latter states and the ground vibrational state of neutral water. The agreement with the experimental values is reasonably good.^50,51^ In particular, the difference between calculated and measured energies is typically of the order of 5 meV. Table III shows a comparison of our calculated FC factors with those reported previously but referred to the (0, 0, 0) level^50,52^ (no absolute values were given in the latter works). The agreement is also reasonably good. As expected from the above discussion, the largest FC overlap is found for the (0, 0, 0) state of the cation, followed by those involving the (1, 0, 0) and (0, 1, 0) vibrational states. The FC overlaps with the (0, 0, 1) and higher $\left(0,0,v_{\mathrm{as}}^{\prime }\right)$ states (except for $v_{\mathrm{as}}^{\prime }=2$) are negligible because, along the antisymmetric stretching normal-mode coordinate, the PESs of the ^1^A_1_ and ^2^B_1_ states are nearly parallel and almost perfectly harmonic so that the initial (0, 0, 0) state is practically orthogonal to them. The (0, 0, 2) state is an exception, since, as mentioned above, it is strongly coupled to the neighboring (2, 0, 0) state. As we will see below, this “accidental” quasidegeneracy will manifest in both the calculated and measured photoelectron spectra.

**TABLE III.** Relative Franck-Condon factors of the vibrational states associated with the ^2^B_1_ ground electronic state of H_2_O^+^. Vibrational configuration interaction (VCI): Theoretical results from Ref. 52 using the vibrational configuration interaction approach. Experimental values taken from Ref. 50. Notations as in Table II.

$\left(v_{\mathrm{ss}}^{\prime },v_{b}^{\prime },v_{\mathrm{as}}^{\prime }\right)$	This work	VCI theory^52^	Exp.^50^
(0,0,0)	1.000	1.000	1.000
(0,1,0)	0.100	0.107	0.089
(0,2,0)	0.014	0.012	0.010
(1,0,0)	0.189	0.200	0.193
(0,0,1)	0.000	0.000	…
(0,3,0)	0.001	0.001	0.003
(1,1,0)	0.017	0.020	0.016
(0,1,1)	0.000	…	…
(0,4,0)	0.000	…	…
(0,2,1)	0.000	…	…
(1,2,0)	0.003	0.003	0.003
(1,0,1)	0.000	…	…
(2,0,0)	0.021	0.022	0.023
(0,0,2)	0.007	0.007	…
(0,5,0)	0.000	…	…
(0,3,1)	0.000	…	…
(1,3,0)	0.000	…	0.002
(1,1,1)	0.000	…	…
(2,1,0)	0.001	0.002	…

**FIG. 3.**
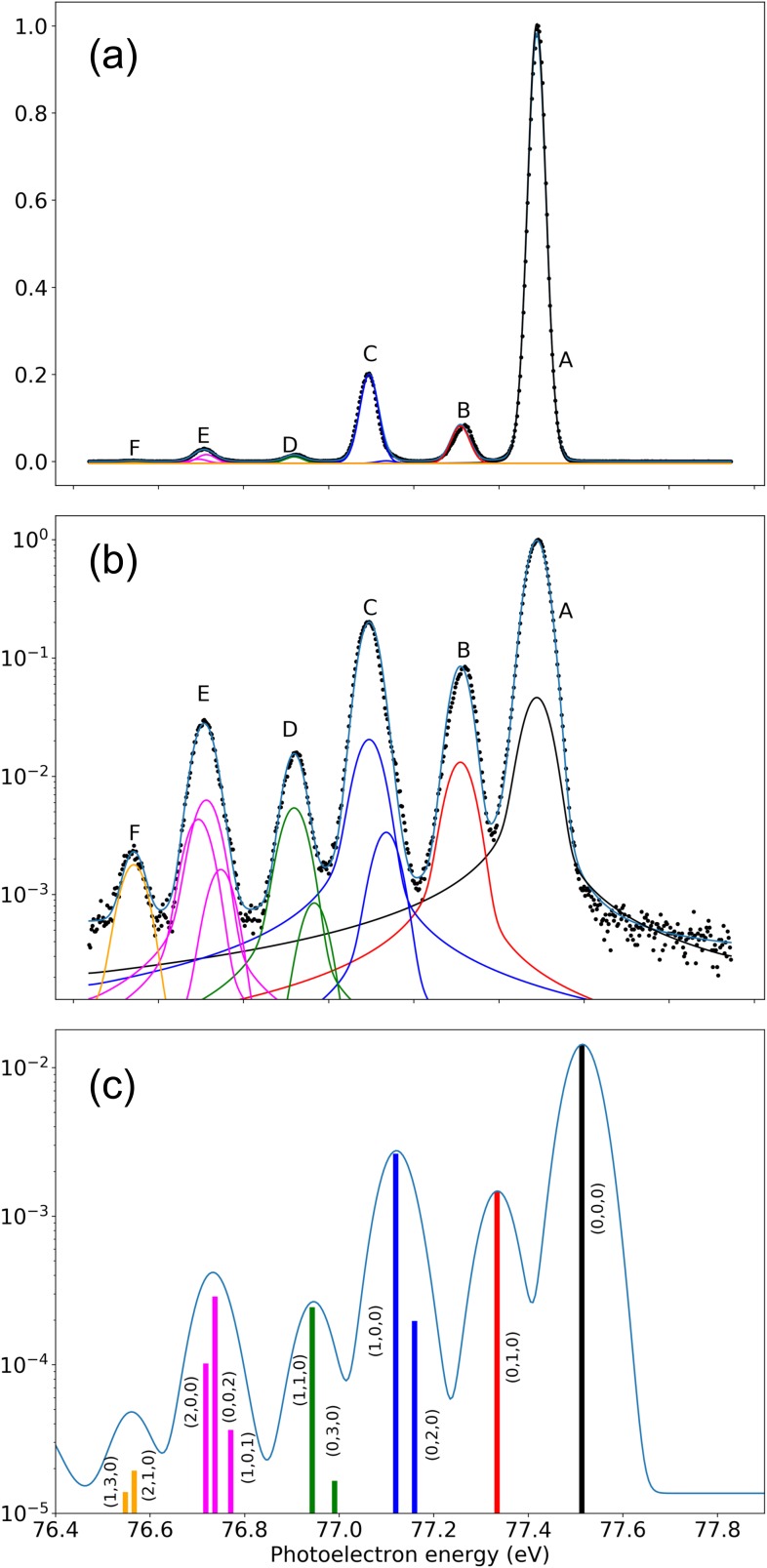
Photoelectron spectrum of H_2_O (^1^A_1_) leading to H_2_O^+^(^2^B_1_) at a photon energy of 91 eV. (a) Experimental result. (b) Experimental result on a logarithmic scale. (c) Theoretical results: with infinite energy resolution (vertical bars) and convoluted with the experimental energy resolution (on a logarithmic scale). The label $\left(v_{b}^{\prime },v_{\mathrm{ss}}^{\prime },v_{\mathrm{as}}^{\prime }\right)$ indicates the vibrational quantum numbers in the bending, symmetric stretching, and antisymmetric stretching modes corresponding to the dominant component in the vibrational eigenvector.

Figure 3 shows the measured photoelectron spectrum for ionization of H_2_O (^1^A_1_) to H_2_O^+^(^2^B_1_) at a photon energy of 91 eV. The spectrum is shown on both normal [panel (a)] and logarithmic [panel (b)] scales. In order to identify the different vibrational sequences in the measured spectra, the latter were fitted by using the Igor Pro data analysis software from WaveMetrics, Inc., and the SPANCF fitting macros by Kukk *et al.*,^5,53^ following a procedure similar to that described in Ref. 17. In particular, postcollisional interaction line shapes^54,55^ were included by using a Lorentzian function with a common width for all the lines as a free parameter. The Gaussian width representing the instrumental and translational Doppler broadenings was also kept as a free parameter, but constrained to be the same to all peaks in the spectra.

The calculated spectrum is shown in panel (c) of Fig. 3. As can be seen, the agreement between theory and experiment is very good, even for the peaks whose intensities are less than 1% of the dominant peak. In agreement with the above discussion, the dominant peak (denoted A in the figure) corresponds to the (0, 0, 0) vibrational state, followed by the (1, 0, 0) and (0, 1, 0) ones. In the experiment, the (1, 0, 0) peak cannot be separated from the (0, 2, 0) one. Convolution of the calculated spectrum with a Gaussian function of 0.08 eV fwhm that accounts for the limited energy resolution of the experiment leads to a single, slightly antisymmetric peak (denoted C in the figure) with a relative height very similar to that found experimentally. Experimental peaks D, E, and F also result from the contribution of several final vibrational states. In all cases, the agreement with the convoluted theoretical spectrum is reasonably good. In particular, the form of peak E reflects the relatively strong interaction between the (2, 0, 0) and (0, 0, 2) vibrational modes mentioned above. The former interaction leads to an energy separation larger than the one between the corresponding uncoupled harmonic modes and together with the (1, 0, 1) mode manifests as a shoulder in the high energy part of the peak, in good agreement with the experimental observations. It is worth noticing that the relative intensities of the calculated peaks are very similar to the relative intensities of the corresponding FC overlaps, since in the small energy interval considered in Fig. 3, the dipole matrix elements connecting the initial electronic state and the final continuum state barely change with energy. This is by the way the reason why, in most previous works, the analysis of photoelectron spectra obtained at a given photon energy has been performed by comparing with calculated FC factors only.

**FIG. 4.**
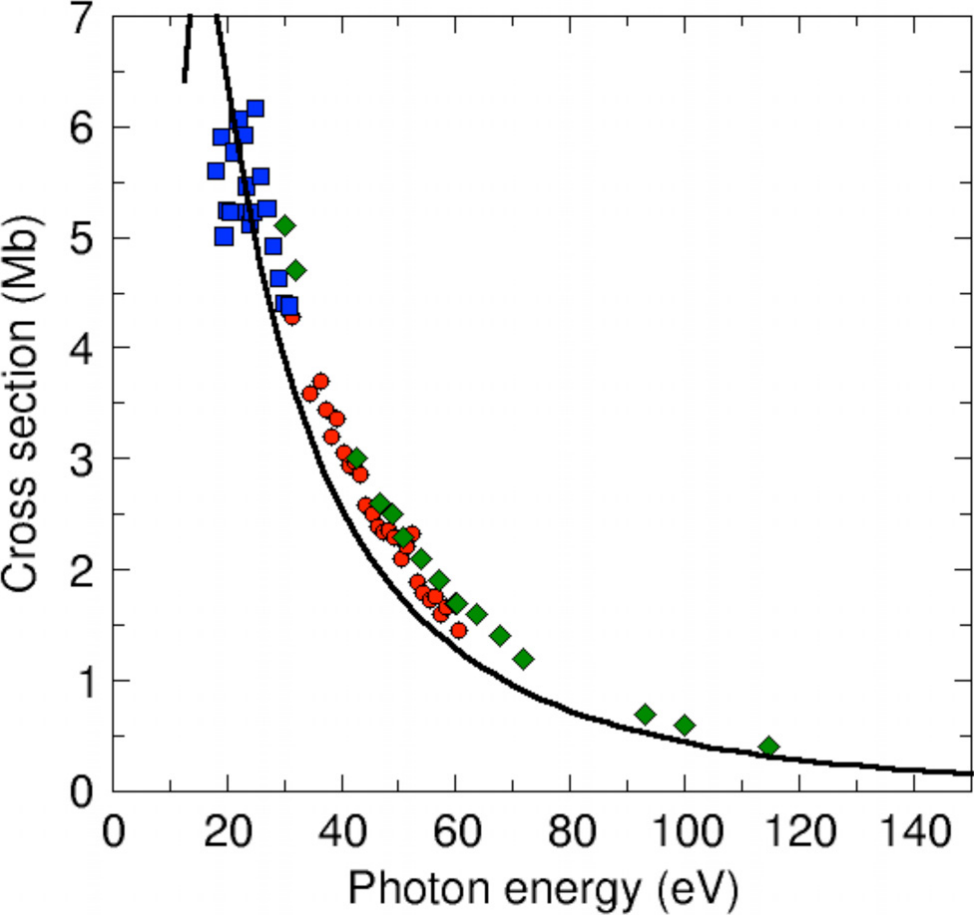
Total photoionization cross sections as a function of photon energy for ionization leading to H_2_O^+^(^2^B_1_). Line: theoretical results. Circles, squares, and diamonds: experimental results from Refs. 56, 57, and 58, respectively.

Figure 4 shows a comparison of the calculated vibrationally unresolved photoionization cross section for the H_2_O^+^(^2^B_1_) channel with that of earlier experimental measurements.^56–58^ No rescaling of the calculated or measured cross sections has been applied. It can be seen that, although the theoretical values slightly underestimate the experimental ones over the whole energy range investigated here, the general agreement is qualitatively good. As mentioned above, the cross section exhibits the typical exponential decrease with photon energy, which prevents one from identifying diffraction features that could be used for structural determination. The remaining discrepancies with experiment are probably due to small deficiencies in the description of the bound states of the neutral molecule and the cation by the present DFT approach. Indeed, the use of more sophisticated methods for the description of these states, as for example coupled-cluster theory,^46^ can lead to even better agreement with experiment. However, to date, these methods have only been used to obtain photoionization cross sections at the equilibrium geometry.

**FIG. 5.**
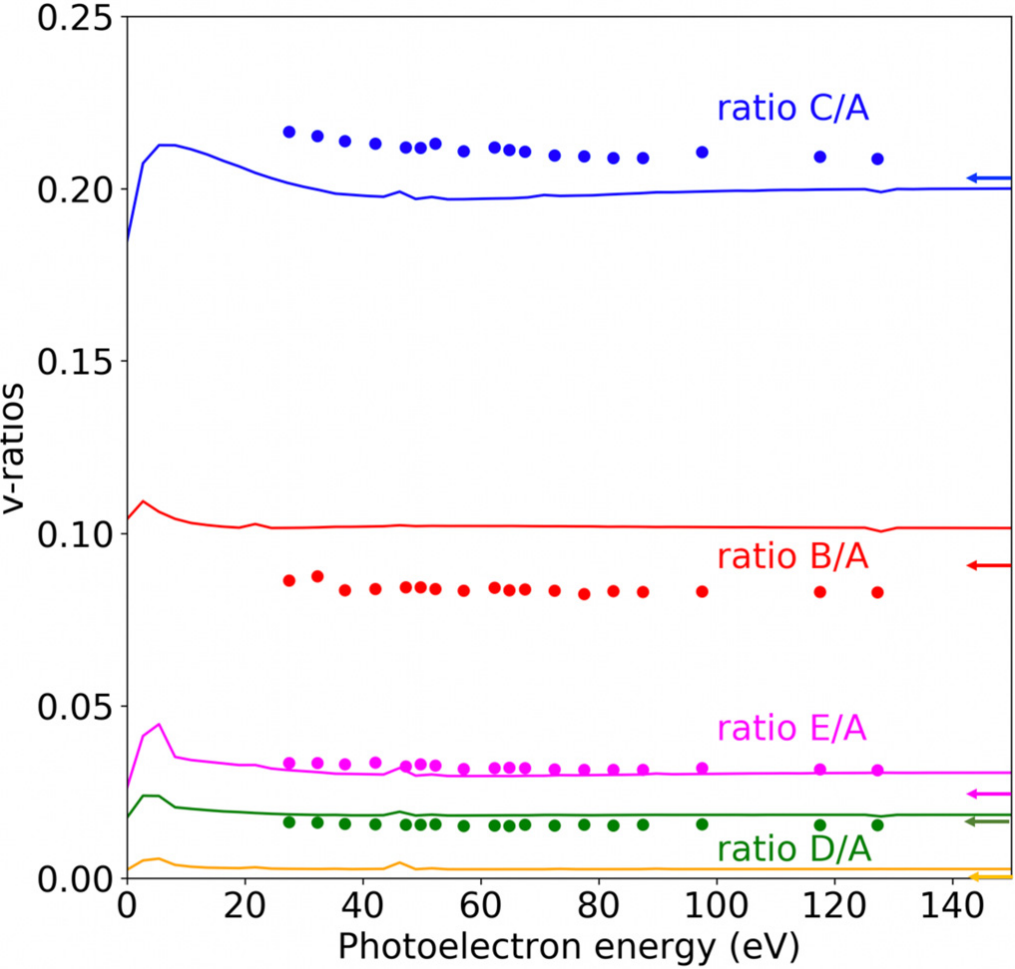
*v*-ratios as a function of photoelectron energy for H_2_O (^1^A_1_) photoionization leading to H_2_O^+^(^2^B_1_). Full symbols: experimental results. Lines: theoretical results. Horizontal arrows indicate the ratios obtained from the corresponding experimental FC factors reported in Ref. 50.

Figure 5 shows the calculated and measured *B*/*A*, *C*/*A*, *D*/*A*, and *E*/*A* ratios as a function of photoelectron energy for ionization of H_2_O (^1^A_1_) leading to H_2_O^+^(^2^B_1_). Among them, only the *B*/*A* ratio corresponds to a true *v*-ratio, namely, the (0, 1, 0)/(0, 0, 0) one. The others are combinations of *v*-ratios, e.g., *C*/*A* = [(1, 0, 0) + (0, 2, 0)]/(0, 0, 0). The agreement between calculated and measured ratios is good, except for the magnitude of the *B*/*A* and *C*/*A* ratios. These differences in magnitude between theoretical and experimental results can be due to inaccuracies in the calculated PESs (see below) or to small errors in the extraction of the individual peak intensities from experiment. In any case, the observed trends are identical. The calculated vibrationally resolved cross sections (not shown) exhibit a maximum just above the ionization threshold, with some additional structures that are due to shape resonances, and beyond these maxima, they decrease exponentially. As the rate of this decrease is very similar for all final vibrational states, the ratios shown in Fig. 5 are nearly flat. In contrast, for centrosymmetric molecules, quite a few number of experimental and theoretical works have reported noticeable oscillations superimposed to a flat background.^13,15–18^ As described above, these oscillations are the signature of photoelectron diffraction by the various atomic centers. In the present case, however, Fig. 5 does not show any clear evidence of oscillations, thus suggesting that intramolecular diffraction effects may not be as important in valence shell photoionization of water.

To get a deeper insight into this matter, we analyze now the individual *v*-ratios. At high photoelectron energy, the values of the *v*-ratios are very close to those of the corresponding ratios between FC factors. Indeed, in this region, the ionization amplitudes vary very slowly with photoelectron energy, and therefore, the photoionization cross section given by Eqs. (1)–(3) can be written to a good approximation as

$$\sigma_{\alpha }\left(v\prime ,\omega \right)=\frac{4\pi^{2}\omega }{3\hbar c}a_{0}^{2}\;F(\alpha ,v\prime )\sum_{\eta }\sum_{l_{\eta }}{\left|\mu_{\alpha_{\eta }l_{\eta }}\left(\varepsilon ,Q_{\mathrm{eq}}\right)\right|}^{2},$$ (5)

where

$$F(\alpha ,v\prime )={\left|\int \chi_{M,v_{0}}^{*}\left(Q\right)\chi_{M_{\alpha }^{+},v^{\prime }}\left(Q\right)dQ\right|}^{2}$$ (6)

is the FC factor connecting the initial vibrational state and the final vibrational state $v\prime =(v_{\mathrm{ss}}^{\prime },v_{b}^{\prime },v_{\mathrm{as}}^{\prime })$ and **Q**_*eq*_ represents the coordinates of the nuclei at the equilibrium geometry. Under this approximation, the *v*-ratios are simply given by the expression

$$\frac{\sigma_{\alpha }\left(v_{a}^{\prime },\omega \right)}{\sigma_{\alpha }\left(v_{b}^{\prime },\omega \right)}=\frac{F(\alpha ,v_{a}^{\prime })}{F(\alpha ,v_{b}^{\prime })}.$$ (7)

This equation shows that the magnitude of the *v*-ratios at high energy is dominated by the magnitude of the FC ratios. So small errors in the values of the latter can lead to appreciable upward or downward shifts with respect to the measured *v*-ratios. In fact, by using the slightly more accurate experimental values of the FC factors previously reported in the literature^50^ (see horizontal arrows in Fig. 5 and Table III), one would obtain a better agreement with the measured B/A and C/A ratios at the highest energies, although there would still remain visible discrepancies.

To better visualize deviations from the FC behavior at the lower energies, where diffraction effects are expected to be more visible, it is more convenient to normalize the *v*-ratios to the FC ratios as follows:

$$\frac{\sigma_{\alpha }\left(v_{a}^{\prime },\omega \right)/F(\alpha ,v_{a}^{\prime })}{\sigma_{\alpha }\left(v_{b}^{\prime },\omega \right)/F(\alpha ,v_{b}^{\prime })}=\frac{\sigma_{\alpha }\left(v_{a}^{\prime },\omega \right)}{\sigma_{\alpha }\left(v_{b}^{\prime },\omega \right)}\times \frac{F(\alpha ,v_{b}^{\prime })}{F(\alpha ,v_{a}^{\prime })}.$$ (8)

**FIG. 6.**
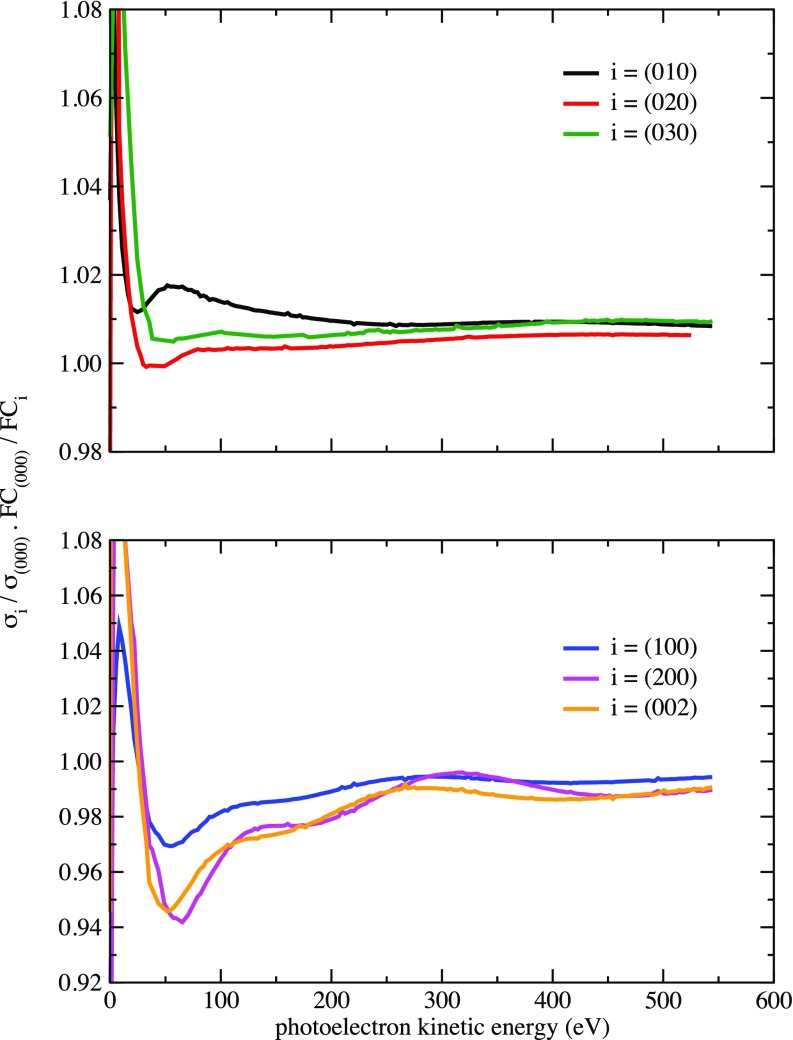
Calculated normalized *v*-ratios defined in Eq. (8) as functions of photoelectron energy for H_2_O(^1^A_1_) photoionization leading to H_2_O^+^(^2^B_1_).

In the FC approximation, the normalized *v*-ratios are equal to 1 for all final vibrational states, and so any deviations from this approximation can be easily identified. Figure 6 shows the normalized *v*-ratios for a few $\left(v_{\mathrm{ss}}^{\prime },0,0),\;(0,v_{b}^{\prime },0\right)$, and $\left(0,0,v_{\mathrm{as}}^{\prime }\right)$ final vibrational states. For the former and the latter, the figure shows the presence of oscillations with very small amplitude. These oscillations are undoubtedly due to diffraction of the ejected photoelectron by the water atomic centers and, therefore, contain structural information on the water molecule. The oscillations are much less visible for the bending mode. The superposition of the bending and symmetric stretching vibrational peaks in the experimental spectrum (e.g., peak C in Fig. 3) together with the weak diffractive power of the swift H atoms makes the observation of these oscillations very difficult. These difficulties could only be overcome by substantially increasing the energy resolution and improving the statistics of the measurements.

## V. CONCLUSION

We have calculated valence-shell vibrationally resolved photoelectron spectra of the water molecule by using an extension of the static-exchange DFT method that incorporates the nuclear degrees of freedom in full dimensionality. To check the validity of the method, we have measured the corresponding *v*-ratios at the SOLEIL synchrotron. The good agreement between theory and experiment validates the theoretical treatment and shows that the bending and the two stretching modes (symmetric and antisymmetric) play a crucial role in the measured spectra. These spectra also reflect the couplings between some of these modes. We have found that diffraction of the ejected electron by the various atomic centers is barely visible in the *v*-ratios, in contrast to the prominent oscillations observed in K-shell ionization of centrosymmetric molecules. One can see, however, a reminiscence of these oscillations when the *v*-ratios are normalized to the ratios between the corresponding FC factors. For their experimental observation, a significant improvement of statistics in the high photon energy region is indispensable, which is difficult due to the smallness of the photoionization cross sections in that region, as well as an improvement in energy resolution that allows one to separate individual vibrational peaks in the photoelectron spectra.

In recent work, the presence of oscillations in the *v*-ratios has been exploited to extract bond distances from neutral and singly ionized species using different fitting procedures. This was facilitated by (i) the initial localization of the ejected electron, as, e.g., in single-center K-shell ionization of centrosymmetric molecules, in which the electron ejected from the central atoms is diffracted by the equivalent peripheral atomic centers, or (ii) the initial delocalization of the ejected electron over two identical centers, as, e.g., in two-center K-shell ionization of homonuclear diatomic molecules or acetylene. In the present work, however, the smallness of the oscillation amplitudes together with the fact that all vibrational normal modes contribute and some of them are significantly coupled makes extraction of bond distances a very challenging problem. Thus, this work calls for the development of more sophisticated fitting models that allow one to extract structural information from valence-shell photoionization of molecules by including contribution from several or all vibrational normal modes and the coupling between them.
